# Comparison between single and multi-locus approaches for specimen identification in *Mytilus* mussels

**DOI:** 10.1038/s41598-019-55855-8

**Published:** 2019-12-23

**Authors:** María Angélica Larraín, Pía González, Claudio Pérez, Cristián Araneda

**Affiliations:** 1 Food Quality Research Center, Universidad de Chile, Santiago, Chile; 2 Laboratorio de Genética y Biotecnología en Acuicultura, Departamento de Producción Animal, Facultad de Ciencias Agronómicas, Universidad de Chile, Santiago, Chile; 3 Departamento de Ciencia de los Alimentos y Tecnología Química, Facultad de Ciencias Químicas y Farmacéuticas, Universidad de Chile, Santiago, Chile; 4 Programa de Magister en Alimentos. Mención Gestión, Calidad e Inocuidad de los Alimentos. Facultad de Ciencias Químicas y Farmacéuticas, Universidad de Chile, Santiago, Chile

**Keywords:** Genetic markers, Haplotypes

## Abstract

*Mytilus* mussels have been the object of much research given their sentinel role in coastal ecosystems and significant value as an aquaculture resource appreciated for both, its flavour and nutritional content. Some of the most-studied *Mytilus* species are *M. edulis*, *M. galloprovincialis*, *M. chilensis* and *M. trossulus*. As species identification based on morphological characteristics of *Mytilus* specimens is difficult, molecular markers are often used. Single-locus markers can give conflicting results when used independently; not all markers differentiate among all species, and the markers target genomic regions with different evolutionary histories. We evaluated the concordance between the PCR-RFLP markers most commonly-used for species identification in mussels within the *Mytilus* genus (*Me15-16*, *ITS*, *mac-1*, *16S rRNA* and *CO*I) when used alone (mono-locus approach) or together (multi-locus approach). In this study, multi-locus strategy outperformed the mono-locus methods, clearly identifying all four species and also showed similar specimen identification performance than a 49 SNPs panel. We hope that these findings will contribute to a better understanding of DNA marker-based analysis of *Mytilus* taxa. These results support the use of a multi-locus approach when studying this important marine resource, including research on food quality and safety, sustainable production and conservation.

## Introduction

Marine mussels within the *Mytilus* genus are benthic organisms inhabiting the intertidal temperate and cold waters of both hemispheres^1,2^. As of April 2019, the Integrated Taxonomic Information System (ITIS) (http://www.itis.gov)^3^ listed four taxa within the genus: *M. edulis* Linnaeus,1758; *M. galloprovincialis* Lamarck, 1819; *M. californianus* Conrad, 1837 and *M. trossulus* Gould, 1850. The World Register of Marine Species (http://www.marinespecies.org)^4^ includes the above plus four additional taxa: *M. planulatus* Lamarck, 1819; *M. platensis* d’Orbigny, 1842; *M. chilensis* Hupé, 1854 and *M. unguiculatus* Valenciennes, 1858. As genetic similarity among these taxa is high, mussels interbreed when they coexist spatially, forming hybrid zones that have been studied by various authors, e.g. Crego-Prieto *et al*.^5^, Inoue *et al*.^6^, Kartavtsev *et al*.^7^, Mathiesen *et al*.^8^, Oyarzún *et al*.^9^, Rawson *et al*.^10^, Riginos & Cunningham^11^, Väinölä & Hvilsom^12^ and Wilhelm & Hilbish)^13^.

*Mytilus* bivalves are an object of research as cosmopolitan inhabitants of high-latitude coastal marine ecosystems in the Northern and Southern Hemispheres, serving as sensitive pollution bioindicators with great utility in ecotoxicology^14–16^. *Mytilus* bivalves are also of interest in invasion ecology, with *M. galloprovincialis* listed among the 100 most invasive species in the world^17,18^. Finally, mussels are highly valued as a flavorful and nutritious food. Mussels are extensively cultured and commercialized in many countries, representing an important economic activity for coastal communities. The FAO reported that the smooth-shelled blue mussel species *M. chilensis, M. edulis* and *M. galloprovincialis* represented 91.8% of *Mytilus* landings worldwide in 2016^19^.

Species-level identification based on morphological traits is problematic within the *Mytilus* genus^20^, as shell shapes are fairly uniform and show environmentally-influenced phenotypic plasticity^6,21,22^. Furthermore, the shell is typically removed from processed foods, further hampering appearance-based identification^23^. Alternatively, mussels are characterized using genetic markers, with approaches relying on allozymes^24–27^, sequencing of mitochondrial genes^2,28–30^ and PCR-based DNA markers, such as amplified fragment length polymorphisms (AFLP)^22^, random amplification of polymorphic DNA (RAPD)^31^, forensically informative nucleotide sequencing (FINS)^23^ and fragment length polymorphisms (FLP). To enhance the specificity of FLP analysis, an enzymatic restriction step can be added to produce restriction fragment length polymorphism (RFLP) markers. Some RFLP banding patterns are easy to score and are almost fixed in allopatric populations. Therefore, these patterns are regarded as diagnostic loci and are widely used for *Mytilus* species identification^32–34^. Nowadays, is also possible to perform a trustworthy specimen identification with highly informative SNP panels^35–40^. However, no all laboratories have access to this genomic technology and PCR-RFLP markers are still used in recently valuable studies^41–44^.

PCR-RFLP analyses of mitochondrial loci have targeted genes such as the large 16S subunit of the rRNA gene^45,46^, the control region of maternally transmitted mtDNA and the cytochrome oxidase subunit I region (*CO*I)^20^ of maternally transmitted mtDNA^47^. Nuclear DNA restriction FLP in anonymous and coding regions have been developed for species identification^48^, targeting at least seven nuclear loci: The internally transcribed spacer regions between the 18S and 28S rDNA nuclear coding regions (*ITS*)^33^, the protamine-like sperm packaging protein (*PLIIa*)^33^, an intron-length polymorphism at the actin gene locus (*mac-1*)^49^, a length polymorphism in the elongation factor 1 cDNA (*EFbis*)^50^, a coding locus designated as *Mytilus* anonymous locus-I (*MAL-*I)^34,51^, the acrosomal sperm protein M7 lysin^52,53^ and the polyphenolic adhesive protein gene, which encodes for a highly-conserved protein that allows mussels to adhere to bedrock.

The polyphenolic adhesive protein gene has been widely used for *Mytilus* species identification. Inoue *et al*.^54^ developed the *Me15-16* primer set for genetic identification of three species (*M. edulis, M. trossulus* and *M. galloprovincialis*). Santaclara *et al*.^23^ added a restriction step to differentiate *M. chilensis* from the Northern-Hemisphere *M. galloprovincialis*. Rawson *et al*.^32^ described two markers: *Glu-5*′, to identify the three Northern Hemisphere blue mussel species, *M. edulis*, *M. galloprovincialis* and *M. trossulus*, and *Glu-3*′, which distinguishes *M. edulis* from *M. galloprovincialis*. Fernández-Tajes *et al*.^20^ used the primers *Myti*-F/R and further digestion with restriction enzymes (*Aci* I and *Acl* I) to differentiate commercial *Mytilus* species, while Jilberto *et al*.^55^ used the primers PAPM F/R followed by high-resolution melting analysis to differentiate *M. chilensis M. edulis* and *M. galloprovincialis* and their hybrids. Interestingly, *Me15-16*, *Glu-5*′*, Myti* and PAPM target the same region in the gene.

According to a search of the Science Direct and Web of Science databases for research using any PCR-RFLP DNA analysis to identify *Mytilus* species published from 1995 to date, ~80% of studies used a marker that targets the polyphenolic adhesive protein gene. Most works (~55%) used a single-marker diagnostic test, while others used two (~20%) or three (~20%), and a few (~5%) applied four or more markers. However, in studies with multiple markers, results sometimes varied by marker, as is showed in *Mytilus* populations from Europe^56–59^, Tasmania and the Kerguelen Islands^60,61^ and the Pacific North American coast^62^. These findings have led to divergent descriptions of the species distribution, especially in the Southern Hemisphere. Therefore, applying a combination of markers has been proposed as a more reliable identification method^58^.

Our aim was to compare the performance in *Mytilus* specimen identification obtained using single- and multi-locus approaches, and also to evaluate concordance among the PCR-RFLP markers most commonly used for *Mytilus* mussel identification (*Mytilus* spp.): *Me15-16*, *ITS*, *mac-1*, *16S rRNA* and *CO*I to contribute to a better understanding of the previous works (pre-genomic era) based mostly in the application of mono-locus PCR- RFLP markers to identify *Mytilus* taxa. Considering the irruption of genomic markers (SNPs), we also aim to contrast the performance in specimen identification of multi-locus panels composed by SNPs used by Larraín *et al*.^38^ with the five above mentioned PCR-RFLP markers.

## Methods

### Sampling and DNA extraction

**Figure 1:**
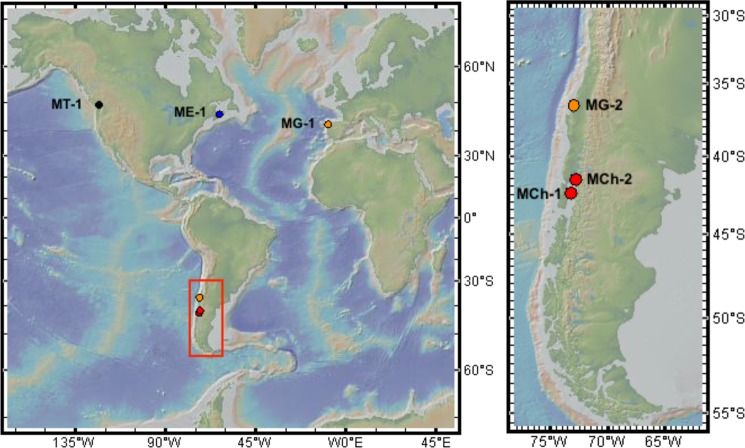
Locations and codes for the six sampling sites. Codes for locations can be found in Table S5. Color indicates species as determined using the PCR-RFLP *Me15-16 Aci*I assay: red for *Mytilus chilensis*, orange for *Mytilus galloprovincialis*, blue for *Mytilus edulis* and black for *Mytilus trossulus*. Background topographic map from GeoMapApp (http://www.geomapapp.org).

Mussel samples (n = 298) were obtained between 2008 and 2017 from six locations (Fig. 1). Four locations represented putatively pure populations outside of described hybrid zones: pacific mussels (*M. trossulus*) (MT-1) from West Vancouver, Canada (n = 50); blue mussels (*M. edulis*) (ME-1) from the Northwest Atlantic Ocean near Prince Edward Island, Canada (n = 50); Mediterranean mussels (*M. galloprovincialis*) (MG-1) from Galicia along the Atlantic coast of Spain (n = 49) and Chilean mussels (*M. chilensis*) (MCh-1) from Putemun on Chiloé Island (n = 50). The latter three are the most commercialized *Mytilus* species. Two other Chilean populations were sampled from locations where hybrid individuals have been found: Quillaipe (MCh-2) (n = 49) on the Gulf of Reloncaví^63^ and the northern coast of the Gulf of Arauco (MG-2) (n = 50), where the Mediterranean mussel (*M. galloprovincialis*) has also been found^30,64^. Detailed sample data is included in Supplementary Information (Table S1). Approximately 50–100 mg of ethanol-fixed mantle edge tissue was used for DNA extraction with a modified phenol-chloroform method^63^. Extracted DNA was quantified with a NanoDrop 2000 spectrophotometer (Thermo Fisher Scientific).

### Specimen identification

**Figure 2:**
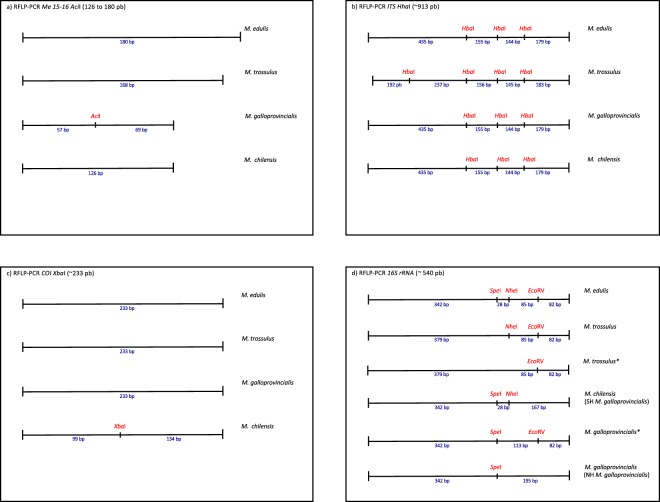
Restriction map of markers RFLP-PCR (**a**) *Me15-16 Aci*I, (**b**) *ITS Hha*I, (**c**) *CO*I *Xba*I and (**d**) *16S rRNA EcoR*V, *Nhe*I and *Spe*I. *Is used to identify the new haplotypes found in this work. For clarity, we will conserve the name *M. galloprovincialis* to refer the former Northern Hemisphere haplotype and use *M. chilensis* for the former Southern Hemisphere haplotype.

To avoid the unintended presence of other genera (i.e. *Aulacomya* or *Choromytilus*) in the Chilean samples, genus assignment was performed with PCR-RFLP using *18S rDNA* and the enzyme *BsaH*I^23^. Species identification was performed for *Mytilus* specimens with PCR-RFLP using the nuclear DNA marker *Me15-16* and the enzyme *Aci*I^23,54^. This assay targets the polyphenolic adhesive protein gene, producing PCR products 180 bp in length for *M. edulis*, 168 bp for *M. trossulus* and 126 bp for both *M. chilensis* and *M. galloprovincialis*. To differentiate between these last two species, amplicons were digested with *Aci*I, producing 77 and 49 bp fragments in *M. galloprovincialis* and leaving the *M. chilensis* amplicon uncut^23^ (Fig. 2a). These classifications were used as the reference for comparisons with the markers below.

The multi-allelic marker nuclear locus *mac-1* has a size polymorphism^49^, allowing for discrimination among *M. edulis, M. trossulus*, and *M. galloprovincialis* according to the frequency of “synthetic” (pooled) alleles^60,65,66^.

The nuclear marker PCR-RFLP *ITS Hha*I produces a ~913 bp fragment. After fragment restriction with *Hha*I endonuclease, the RFLP patterns from *M. edulis, M. galloprovincialis* and *M. chilensis* consisted of four fragments with modal sizes of 435, 179, 155 and 144 pb, respectively, with only minor variations in size by species. Five fragments were observed for *M. trossulus*, with modal sizes of 237, 192, 183, 156 and 145 (Fig. 2b). As in Heath *et al*.^33^ and Toro^67^, this marker therefore only differentiated between *M. trossulus* and the other three species, although those authors obtained somewhat different fragment sizes using agarose gels.

Mitochondrial marker PCR-RFLP *CO*I *Xba*I targets the cytochrome oxidase subunit I region with the primers COIXbaF and COIXbaIR, designed to distinguish *M. chilensis* from other mussels. The 233 bp amplicon was restricted with the *Xba*I enzyme, generating two fragments (134 and 99 bp) in *M. chilensis*^20^ only (Fig. 2c).

The mitochondrial marker PCR-RFLP *16S rRNA* produces a ~540 bp fragment with universal primers 16sar-L/16sbr-H^68^. After digestion with the enzymes *EcoR*V, *Nhe*I and *Spe*I, *M. edulis* (342, 85, 82 and 28- bp fragments) and *M. trossulus* (379, 85 and 82 bp fragments) showed fixed haplotypes. Northern Hemisphere *M. galloprovincialis* individuals showed an exclusive haplotype (342 and 195 bp fragments) as well as the *M. edulis* haplotype (342, 85, 82 and 28 bp fragments). Westfall *et al*.^46^ and Zardi *et al*.^69^ described a Southern Hemisphere *M. galloprovincialis* haplotype with three fragments (342, 167 and 28 bp) (Fig. 2d). For clarity, we will conserve the name *M. galloprovincialis* to refer the former Northern Hemisphere haplotype and use *M. chilensis* for the former Southern Hemisphere haplotype (Fig. 2d).

DNA amplifications were performed in a Techne TC-412 (Bibby Scientific Ltd, UK) thermocycler and Palm-Cycler^TM^ (Corbett Life Science, Australia) with recombinant Taq DNA polymerase (RBC Bioscience^®^ and Thermo Fisher^®^) using 40 ng of template DNA in a final volume of 25 μL. The PCR primer pairs described were used as reported by the above authors with no modifications. Details regarding the PCR reaction conditions and amplification profiles for each marker are shown in Table 1. All experiments included a negative control (with no template DNA added) and a positive control consisting of template DNA that was previously extracted and successfully amplified by conventional PCR with *Me15-16*^54^. Digestion with *Aci*I (New England Biolabs), *Hha*I and *Xba*I (Thermo Scientific) was performed separately in a final volume of 20 μL, using 15 μL of PCR product and 4, 10 and 10 units of each enzyme with 1x NEB4 and Tango buffers, respectively. Triple digestion with *EcoR*V, *Nhe*I and *Spe*I (New England Biolabs) was performed in the same reaction using 10, 5 and 5 units of each enzyme, respectively, with 1x NEB2 buffer. All incubations were carried out overnight at 37 °C. The size of amplified fragments resolved in PAGE was obtained by log-linear interpolation of the 10 bp DNA ladder (Invitrogen^®^) or HyperLadder V (Bioline^®^) on the gel.

**Table 1:** PCR and enzymatic digestion conditions.

	Marker			
	RFLP-PCR *Me15-16 Aci* I	*mac-1*	RFLP-PCR *ITS Hha* I	RFLP-PCR *CO*I *Xba* I
**Component [concentration]**				
**(**40 ng DNA in 25 µL final reaction volume) MgCl_2_ [mM]	2	1.5	2	1.5
dNTP (each) [µM]	50	60	100	200
Primer F and R (each) [µM]	0.4	0.14	0.2	0.6
Taq [U]	0.75	0.5	1	1.5
**Annealing conditions**				
Temperature [°C]	56	46	55	52
Time [s]	30	30	20	30
**Enzimatic digestion conditions** (20 µL final reaction volume)				
Enzime - [U]	*Aci* I - 4	—	*Hha* I - 10	*Xba* I -10

### Genotyping

Genotypes were scored using polyacrylamide gel electrophoresis (PAGE) (8%) and silver staining for the markers *18S rDNA*, *Me15-16*, *mac-1*, *CO*I and *16S rRNA*, except for MT-1 specimens. In these individuals, *mac-1* and *16S rRNA* were genotyped with a Fragment Analyzer^TM^ instrument (Advanced Analytical Technologies, Ames, IA), using the dsDNA 905 Reagent Kit (35–500 bp) following manufacturer instructions. This kit resolves 2 bp differences in DNA fragments and alleles. The data were normalized to 35 bp and 500 bp lower and upper markers and calibrated to the 75 to 400 bp range using PRO Size 2.0 software (Advanced Analytical Technologies, Inc.). Correspondence between the allele sizes obtained using the two methods was established by constructing an allele ladder, sizing all alleles obtained from polyacrylamide-scored genotypes and genotyping this allele ladder with a Fragment Analyzer^TM^ instrument. Finally, the *ITS* marker was genotyped with the Fragment Analyzer^TM^ in all individuals. Quality was verified by including negative controls in each run and re-genotyping a randomly-selected 5% of individuals.

### Data analysis

#### Mono-locus approach

The mitochondrial markers *CO*I and*16S rRNA* might show two haplotypes, due to the double uniparental mitochondrial inheritance (DUI) observed in mussels, thus somatic cells carry the female and, in less frequency, the male mitochondrial genome possibly giving different haplotypes^70^. To avoid affecting the species identification, individuals with two haplotypes were excluded from further analysis. Mitochondrial markers with only one haplotype were analyzed as homozygous diploid genotypes following Narum *et al*.^71^ Numbers and frequencies of alleles and haplotypes were estimated with the R package strataG^72^.

Individuals were assigned to a species based on each marker separately (nuclear *Me15-16*, nuclear *ITS* and mitochondrial *CO*I) according the allele sizes and patterns defined for each species, as described above (Fig. 2). These markers are considered diagnostic in allopatric populations^7^. In the case of multiallelic markers *mac-1* and *16S rRNA*, species was determined using a leave-one-out (LOO) algorithm with the Bayesian method described by Rannala & Mountain^73^ using GeneClass2 software^74^. Individuals were allocated to a species with an assignment threshold of 0.05. This procedure avoids the subjectivity of pooling species-specific compound alleles into synthetic alleles^75,76^.

Each marker was evaluated through re-allocation analysis with the software package GeneClass2 as described above. A re-assignment was considered correct if it matched the classification by PCR-RFLP *Me15-16 Aci*I. Numbers and percentages of matching and mismatching assignments were determined for each marker.

The concordance between PCR-RFLP *Me15-16 Aci*I and the other markers was evaluated graphically using a heatmap as well as classical diagnostic test performance statistics for each species. (i) Sensitivity (S), calculated as the number of individuals correctly assigned to the species divided by the total number of individuals sampled from that species, reflects how well the test correctly assigns individuals to a species^77^. (ii) Specificity (E), calculated as the number of individuals appropriately excluded from the species divided by the total number of individuals who do not belong to the species, reflects how well the test correctly excludes individuals from a species^78,79^. Sensitivity vs. specificity for each marker in each species was plotted. (iii) Positive likelihood ratio (LR+), calculated as S/(1-E), summarizes how many times more likely it is that individuals of a species will be assigned to the species as compared to specimens of other species^80^. When specificity is 1.0, LR+ will be undefined, therefore, we added 0.5 to all counts in the table to calculate an approximate LR+ value^81,82^. The 95% of confidence intervals (95% CI), to determine if the diagnostics statistics values (S, E and LR+) were significantly different from zero, were estimated with the R package epiR (https://cran.r-project.org/web/packages/epiR/).

#### Multi-locus approach

To visualize the separation of species using four markers (excluding PCR-RFLP *Me15-16 Aci*I) and all five markers simultaneously. Two-dimensional factorial correspondence analysis (2D-FCA) and principal component analysis (PCA) were performed with the R package adegenet^83^. As with the mono-locus approach, the performance of the four and five markers together was evaluated using re-allocation analysis with the software package GeneClass2 (described above). We use the non-parametric Wilcoxon signed rank test to compare the assignment performance of the panel composed by the five RFLP-PCR markers obtained in this study (Table S4f) with that obtained with a 49 SNP panel in 338 mussels: *M trossulus* (17), *M. edulis* (27), *M. galloprovincialis* (105) and *M chilensis* (189), from previous work (Larraín *et al*.^38^) and summarized in Table S2. In both studies, PCR-RFLP *Me15-16 Aci*l was used as reference marker to perform specimen identification.

## Results

All 298 individuals were successfully genotyped with *Me15-16*^23,54^ as pure *M. trossulus* (50), *M. edulis* (50), *M. galloprovincialis* (99) or *M. chilensis* (99), producing the expected allele size (Fig. 2a). *ITS* and *CO*I amplified in all individuals with PCR-RFLP. The loci *mac-1* and *16S rRNA* could not be amplified after two attempts each in five individuals. The global genotyping success rate was 98.32% across populations, with a 100% match rate in re-tested individuals (n = 15). On the other hand, only *16S rRNA* marker showed two mitochondrial haplotypes in seven *M. chilensis* from MCh-1, 30 and three *M. galloprovinciali*s from MG-2 and MG-1 populations, respectively. These 40 individuals were excluded from subsequent analysis, as was described in the data analysis section.

### Mono-locus approach

#### First intron in the Mytilus actin protein gene: nuclear locus mac-1

Locus *mac-1* was polymorphic in all locations, with 27 alleles ranging from 164 to 494 bp in length among individuals (Table S3). Two alleles (255 and 266 bp) were present in all four species and all six locations. Two alleles (303 and 328 bp) were exclusive to *M. galloprovincialis*. The frequency of the 328 bp allele was ten-fold higher in the Southern (0.220) than the Northern Hemisphere (0.021), while the reverse was true for the 303 bp allele. The *M. chilensis* populations (MCh-1 and MCh-2) and *M. edulis* sample from Canada (ME-1) were less diverse, with a maximum of three alleles (255, 266 and 298). *M. trossulus* had numerous private alleles with low frequencies except for two higher-frequency alleles (487 and 494 bp).

**Figure 3:**
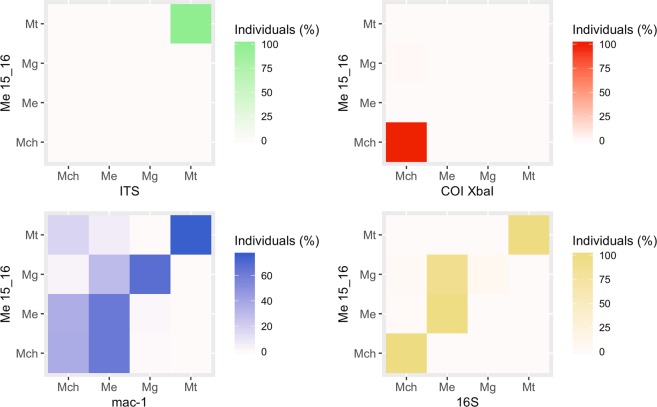
Heatmaps indicating concordance (% of individuals) in species identification between PCR-RFLP *Me15-16 Aci*I and each of the other PCR-RFLP markers evaluated (*ITS, CO*I and *16S rRNA*) and *mac*-1.

When the *mac-1* locus genotypes were used to assign individuals to the species determined by PCR-RFLP *Me15-16 Aci*I, 144 of 253 individuals (56.9%) had matching results for both markers. *mac-1* correctly reassigned 67.7% of *M. galloprovincialis* (44 of 65) but only 37.0% of *M. chilensis* individuals (34 of 92) (Fig. 3, Table S4a), with 57 of 92 individuals (62.0%) from the latter species mis-reassigned to *M. edulis*. For *M. edulis*, 31 of 50 (62.0%) individuals were correctly reassigned, with 18 (36.0%) wrongly classified as *M. chilensis*.

Concordance between *mac-1* and PCR-RFLP *Me15-16 Aci*I is shown in Fig. 3. Sensitivity (number of individuals from each species correctly assigned by *mac-1* divided by total number of individuals from that species) was high for *M. trossulus* and *M. galloprovincialis* (0.76 and 0.68) but only 0.37 for *M. chilensis* (Table S5). This marker accurately excluded individuals from *M*. *trossulus* and *M. galloprovincialis*, with specificities of 1.00 and 0.99, respectively. The positive likelihood ratio indicated that *M. galloprovincialis* individuals were 63.63 times more likely than other individuals to be assigned to the species.

#### Nuclear marker PCR-RFLP ITS HhaI

**Figure 4:**
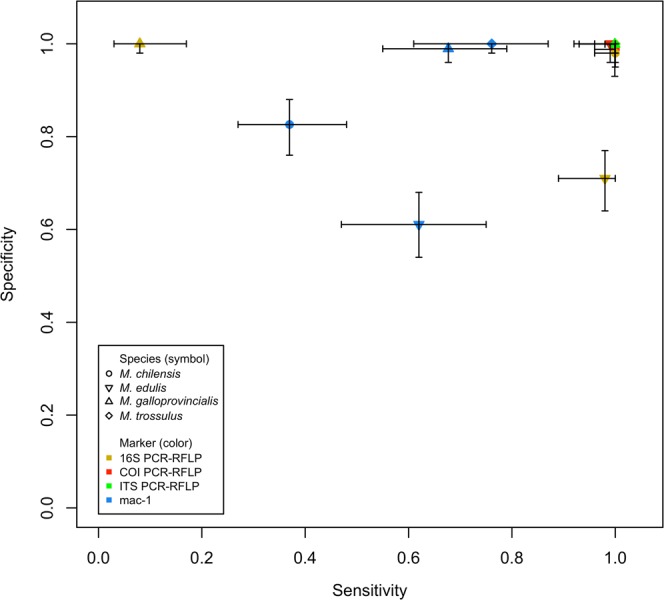
Sensitivity vs. specificity by species and marker, along with the respective 95% confidence intervals (raw data in Table S5). The species are represented by the symbols (°) *Mytilus chilensis*, (∇) *Mytilus edulis*, (Δ) *Mytilus galloprovincialis* and (⬨) *Mytilus trossulus*. The markers are represented by the color: gold for PCR-RFLP*16S rRNA*, red for PCR-RFLP *CO*I, green for PCR-RFLP *ITS*, and blue for *mac*-1.

The RFLP assay clearly differentiated *M. trossulus* from *M. chilensis, M. edulis* and *M. galloprovincialis* but, as expected, did not distinguish among the latter three (Fig. 2b). All 50 *M. trossulus* individuals were correctly re-assigned and the other 208 individuals correctly excluded (Fig. 3, Table S4b). Sensitivity and specificity for *M. trossulus* were optimal, with full concordance between *ITS* and *Me15-16* (Fig. 4, Table S5).

#### Cytochrome oxidase subunit I gene: mitochondrial marker PCR-RFLP COI XbaI

As expected, species identification results matched for *CO*I and *Me15-16* in 100% of *M. chilensis* individuals (MCh-1 and MCh-2), indicating optimal sensitivity (1.00) (Figs. 3 and 4). In two (B12 and B42) of the 50 *M. galloprovincialis* individuals from Dichato, Chile (MG-2), the amplicon was digested, producing the two-fragment pattern characteristic of *M. chilensis* (Fig. 2c. Table S4c), resulting in a specificity of 0.99 for *M. chilensis* (Table S5). This mitochondrial marker did not distinguish among *M. trossulus, M. edulis* and *M. galloprovincialis* but correctly excluded all *M. chilensis* specimens (specificity = 1.00).

#### 16S rRNA gene: mitochondrial marker PCR-RFLP 16S rRNA

After triple enzymatic digestion, we found other two haplotypes not previously described for this locus (shown with asterisk in Fig. 2d). The first one was present in one MT-1 individual (0.022) that was missing the *Nhe*I site in contrast to the standard *M. trossulus* haplotype. The second was present in three MG-2 individuals (0.15), each with the *EcoR*V site present and *Nhe*I site missing in contrast to the standard *M. galloprovincialis* haplotype (Fig. 2d).

The *M. chilensis* haplotype (the species previously named Southern Hemisphere *M. galloprovincialis*) was fixed (1.0) in MCh-1 and MCh-2. The frequency of this haplotype was of 0.10 in the third Chilean population (MG-2), identified as *M. galloprovincialis* by PCR-RFLP *Me15-16 Aci*I. In the Northern Hemisphere populations, this haplotype was present at very low frequencies (0.02) only in ME-1, and absent in MG-1 and MT-1 (Table S6).

As expected, the *M. edulis* haplotype was frequent in ME-1 (0.980) but also in MG-1 (0.957), identified as *M. galloprovincialis* by PCR-RFLP *Me15-16 Aci*I. In this latter population from Galicia, the low frequency of the *M. galloprovincialis* haplotype (0.043) was unexpected (Table S6).

When species identification results for PCR-RFLP *16S rRNA* and PCR-RFLP *Me15-16 Aci*I were compared (Fig. 3, Table S4d), 191 of 253 individuals (75.5%) had matching results. *16S rRNA* correctly reassigned 100, 98 and 100% of *M. trossulus, M. edulis and M. chilensis* individuals respectively, but only 5 of 66 (7.6%) *M. galloprovincialis* individuals. Moreover, 59 of 66 (89.4%) from this latter species were mis-reassigned to *M. edulis* and 2 of 66 (3.0%) to *M chilensis*.

Concordance between *16S rRNA* and *Me15-16* is shown in Fig. 3. The sensitivity of PCR-RFLP *16S rRNA* was optimal for *M. trossulus* and *M. chilensis* (1.00) and high for *M. edulis* (0.98) but very low for *M. galloprovincialis* (0.08) (Fig. 3, Table S3). In general, the method correctly excluded individuals from *M*. *trossulus* and *M. galloprovincialis*, with specificities of 1.00 and also *M. chilensis* (0.98).

### Multi-locus approach

**Figure 5:**
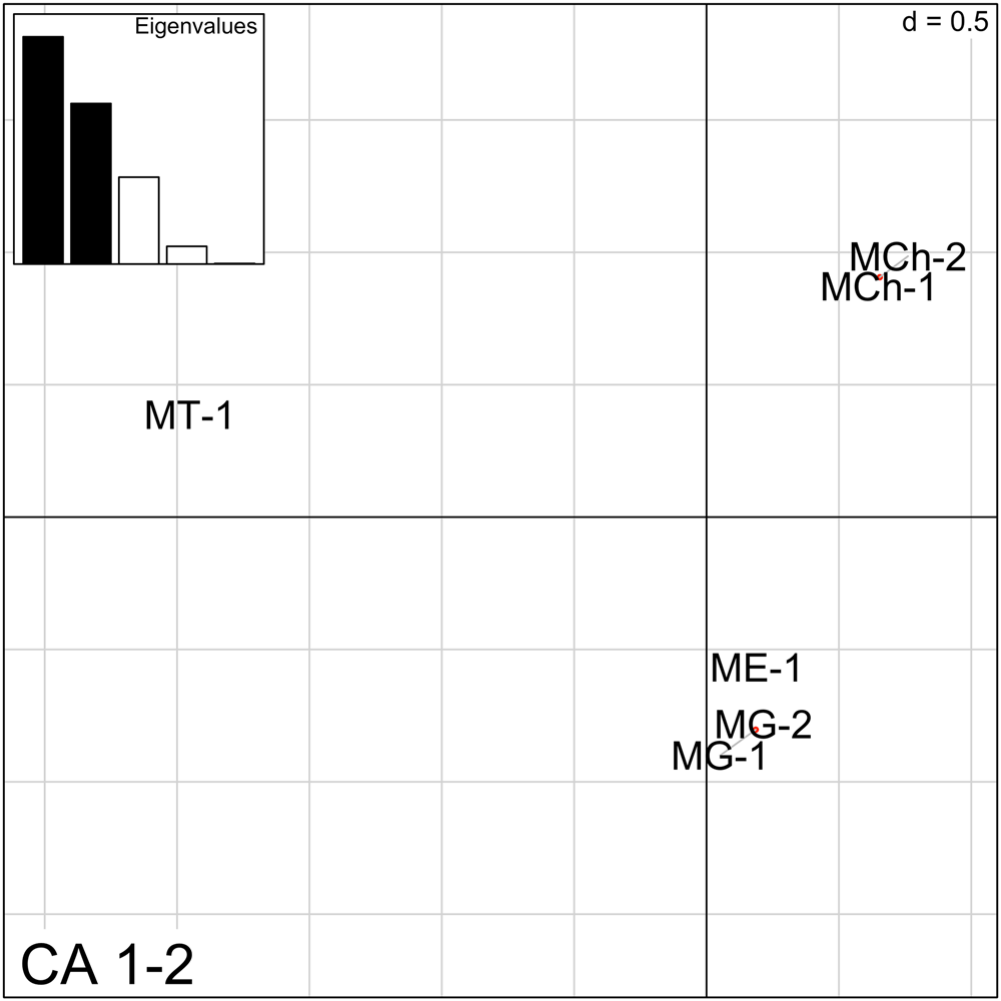
Two-dimensional factorial correspondence analysis (2D-FCA) constructed using the information from all five markers simultaneously. Eigenvalues corresponding to the represented components are filled in black.

**Figure 6:**
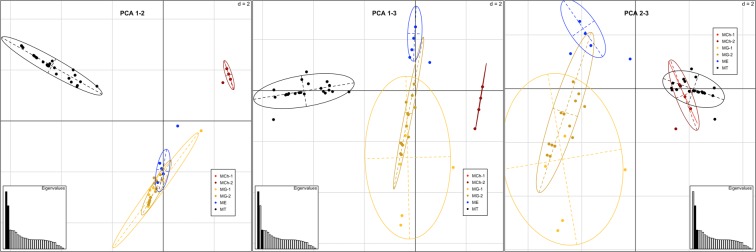
Principal component analysis (PCA) constructed using the information from all five markers simultaneously. Eigenvalues corresponding to the represented components are filled in black. Points represent genotypes and inertia ellipses are placed in 95%. Sampling locations are represented by the colors: red for MCh-1, dark red for MCh-2, blue for ME-1, yellow for MG-1, gold for MG-2 and black for MT-1.

This analysis uses the genetic information provided by all five markers simultaneously. The 2D-FCA multi-locus approach separated all four species, as shown in Fig. 5. As expected, ME-1, MG-1 and MG-2 mapped very closely. The Northern Hemisphere populations (MG-1 and ME-1) overlapped in all of the PCA plots (Fig. 6). These plots also showed an overlap between the Chilean *M. galloprovincialis* population (MG-2) and the same species from Spain (MG-1) but no overlap with ME-1. On the other hand, the *M. chilensis* populations (MCh-1and MCh-2) were clearly separated from the other *Mytilus* species, including *M. trossulus* (MT-1).

Assignments performed using all five markers simultaneously showed 100% concordance with PCR-RFLP *Me15-16 Aci*I (Table S4f). When *Me15-16* was excluded, the percentage of correct species re-assignment remained at 100% for *M. trossulus* and *M. chilensis* but dropped to 96% for *M. edulis* and 65% for *M. galloprovincialis* (Table S4e). Sensitivity remained high when four markers were used (excluding *Me15-16*). Specificity was 1.00 for *M. trossulus* and *M. chilensis*, 0.96 for *M. edulis* but dropped to 0.65 for *M. galloprovincialis* (Table S5).

The comparison of assignment performance obtained with the RFLP-PCR multi-locus panel and the SNP panel did not show significantly differences (*p-valu*e = 1.00), indicating the same performance of both kind of markers sets.

## Discussion

The use of genetic methods to assign taxonomic names to unknown *Mytilus* individuals, called specimen identification^84^, is useful for: increasing genetic knowledge of mussels, studying populations and hybrid zones, establishing taxonomy and systematics, identifying evolutionary relationships and phylogeny within the genus, performing ecological studies and verifying food authenticity.

As the *Mytilus* genus contains several taxa, researchers may need to authenticate the target species using molecular markers. Species assignment is typically performed using a single locus independently, called the mono-locus approach, likely because this method is relatively fast and cheap^85^. However, not all markers can differentiate all of the species in the genus. Furthermore, because the various markers target different regions in the genome, they often produce nonequivalent classification results^38,86^.

The 65 pb intron length polymorphism in the actin gene *mac-1*, used for genotyping in *Mytilus* population studies^49^, systematically fails to amplify in some individuals (~2%), possibly due to mutations in priming sites producing null alleles, as in microsatellites^87^. This phenomenon hinders allele scoring, limiting the accuracy of the mono-locus approach with this marker. *mac-1* correctly excluded *M*. *trossulus* and *M*. g*alloprovincialis* from other species but showed a weak ability to identify the four *Mytilus* species, with only 56.9% of assignments matching the results produced using PCR-RFLP *Me15-16* (Table S4a). The poor performance of *mac-1* is attributable to its limited ability to discriminate between *M. edulis* and *M. chilensis*: 18 of 50 *M. edulis* individuals were assigned to *M. chilensis* and 57 of 92 *M. chilensis* to *M. edulis*. Therefore, several studies that have used this marker in Chilean blue mussels (*M. chilensis*) may have been affected by this bias^2,65^. This nuclear marker has multiple alleles, some of which are shared across *Mytilus* species, and is therefore not fully diagnostic (Table S3). This multiallelic characteristic led others to propose the use of “synthetic alleles,” in which several similarly-sized alleles are pooled for specimen identification^76^. Pooling improved performance, but several of the compound synthetic alleles were still not exclusive to any one species^76^. Because the polymorphism in *mac-1* is located in an intronic region, is quite variable even within a given species, as well as technically difficult to score, limiting performance.

*ITS* targets the internally transcribed spacer sectors between the *18S* and the *28S* genes from the nuclear rDNA coding region. The genomic organization of rDNA consists of a variable number of tandem repeats that is sufficient to provide a DNA template for PCR^88^. *ITS* successfully amplified all individuals and allowed for definitive discrimination of *M*. *trossulus*. Although *ITS* could not separate the other three species, concordance with *Me15-16 Aci*I was optimal for *M. trossulus*. This result is consistent with other works comparing *ITS* to other markers^58^. For example, Toro^67^ discriminated *M. trossulus* from *M. chilensis* and *M. edulis* but could not distinguish between the latter two. In the same work, these three species were clearly differentiated using the *Glu-5*′ marker targeting the *polyphenolic adhesive protein gene*, confirming that *ITS* only can distinguish *M. trossulus* specimens. Due to its multicopy nature, authors have warned that *ITS* should not be considered a codominant single-copy Mendelian marker^56,59^. However, Heat *et al*.^33^ observed a Mendelian-like inheritance pattern when using *ITS* to genotype progeny from two test crosses (*M. edulis* x hybrids *M. edulis*/*M. trossulus*).

The mitochondrial PCR-RFLP *CO*I marker showed full concordance with *Me15-16 Aci*I marker, identifying the Chilean mussel in all locations sampled. However, two individuals (B12 and B42) from the Dichato population (MG-2) in the Arauco Gulf, that were classified as *M. galloprovincialis* by *Me15-16 Aci*I, were identified by *CO*I as *M. chilensis*. The Arauco Gulf is a sympatric zone where the presence of the non-indigenous *M. galloprovincialis* has been described^29,63,64,89,90^. Also, in this zone, our group found a frequency of ~4–7% for hybrids of the two species as part of a routine analysis using the PAPM marker^55^, equivalent to *Me15-16* (unpublished data). The low-frequency presence of individuals carrying the nuclear *M. galloprovincialis* genotype (scored by *Me15-16*) and mitochondrial *M. chilensis* haplotype (scored by *CO*I) shows introgression of the mitochondrial genome from the indigenous *M. chilensis* into the non-indigenous *M. galloprovincialis*, as described in Steinert *et al*.^91^ and Rawson & Hilbish^92^.

The mitochondrial PCR-RFLP *16S rRNA* showed full concordance with the nuclear PCR-RFLP *Me15-16 Aci*I marker for identifying *M. trossulus* and *M chilensis*, but somewhat lower sensitivity for *M. edulis* (0.98). This statistic decreased to 0.08 for the Mediterranean mussel (Table S5, Fig. 4). Most *M. galloprovincialis* specimens were classified as *M. edulis* (59 of 66). This inconsistency between the markers is likely due to the fact that all Mediterranean mussel populations that have been tested in Europe^45^ and the Southern Hemisphere^64,69^ also carry the *M. edulis* haplotype. Therefore, PCR-RFLP *16S rRNA* is only semi-diagnostic for *M. galloprovincialis* and *M. edulis*.

PCR-RFLP *16S rRNA* exhibits an evident DUI of mitochondrial DNA^93^ in 40 samples that present two mtDNA haplotypes, these individuals were not used in further species identification analysis. Of the remaining 20 individuals with one haplotype, 18 had *M. galloprovinciali*s haplotypes and the other two carried the *M. chilensis* haplotype. These last two correspond to the same individuals classified as *M. chilensis* by *CO*I (B12 and B42), as expected by the fact that the mitochondrial genome is considered one locus with *16S rRNA* and *CO*I variants inherited linked. This finding could indicate an asymmetric hybrid zone in the Arauco Gulf area, in which *M. galloprovincialis* being the predominant species, with a lower frequency of the native *M. chilensis*.

Current aquaculture practices in the Arauco Gulf zone involve production of Mediterranean and Chilean mussels in the same area. Interestingly, Westfall & Gardener^64^ also found introgressed individuals with nuclear *M. galloprovincialis* genotypes and mitochondrial *M. chilensis* haplotypes in Cocholgue, a location 12 km away from our sampling point in Dichato, supporting the concept of a hybrid zone.

PCR-RFLP *Me15-16* follows a Mendelian inheritance pattern^94^ and is an extremely robust and reliable diagnostic marker for routine specimen identification^53,95,96^. Therefore, this method is the most common DNA-based technique for identifying mussel species. Of course, *Me15-16* alone is not able to distinguish introgressed individuals^40^, and also, is not able to differentiate *M. chilensis* from the Southern Hemisphere lineage of *M. galloprovincialis* from New Zealand, because in both species, this fragment of genome is not cut by *Aci*I due to the substitution of the allele “G” by “T” in the restriction site^97^.

The mono-locus approach offers some advantages: this method is fast, relatively easy to preform and simple to score with the fully diagnostic markers for the species analyzed here (*Me15-16*, *ITS* and *CO*I). However, they show weakness, such as the fact that some markers have multiple alleles that are not fully fixed in each species (*mac-1* and *16S rRNA*), making them only semi-diagnostic. Also, the presence of multiple alleles sometimes hinders interpretation. Another problem with the mono-locus approach is that some markers cannot identify all of the species analyzed here when used alone, and when two or more are used simultaneously, they produce contradictory results^32,35,61^. Moreover, mitochondrial markers (*CO*I and *16S rRNA*) must be used simultaneously with a nuclear marker to detect introgression in hybrid zones.

The discrepancies observed among the PCR-RFLP markers were expected, as each *Mytilus* species diagnostic marker targets a single locus in distinct zones of the nuclear or mitochondrial genome, likely with different times to common ancestor or gene-genealogy^98^. Moreover, it is widely recognized that evolutionary forces act differently and in an uncoordinated way on nuclear and mitochondrial genomes, and even on different regions within the nuclear genome^11,41^, for example, in monocopy vs. multicopy genes or introns vs. exons. On the other hand, hybridization is also associated with conflicting results, as backcrossing with one or both parental taxa can lead to introgression of alleles from one taxon into the other^99^. In this case, analyses including different types of markers are preferable^41^.

Given that genome of smooth-shelled mussels is about 1.6 Gb^100^, species identification using a single locus or a very small number of loci is relatively straightforward. However, specimen identification based on simultaneous use of the information provided by each locus, known as the multi-locus approach, allows us to consider the evolutionary forces acting on different genomic regions. Therefore, this approach provides more coherent and reliable outcomes, especially when introgression has occurred^58^.

The development of genotyping-by-sequencing methods to affordably discover and genotype hundreds or even thousands of single-nucleotide polymorphism (SNP) markers makes it possible to apply a multi-locus approach to specimen identification^40,101^. New multi-locus SNP panels have been developed recently, allowing for species identification using only the most informative SNPs^38,40,96,102–105^. As an example of the multi-locus approach in mixed populations, hybrids and introgressed individuals were detected by a new twelve-SNP diagnostic panel in European *Mytilus* samples previously analyzed with PCR-RFLP *Me15-16*^40^. The multi-locus approach can also be applied to specimen identification, simultaneously analyzing the mitochondrial and genomic markers traditionally used in mono-locus analysis^58^.

In this work, we used a multi-locus strategy with two mtDNA (PCR-RFLP *CO*I and PCR-RFLP *16S rRNA*) and three nuclear (PCR-RFLP *ITS, mac-1* and PCR-RFLP *Me15-16*) markers. The re-assignment analyses using the multi-locus panel, excluding the reference marker PCR-RFLP *Me15-16*, incorrectly classified 31.8% of *M. galloprovincialis* as *M. edulis*. This finding is not wholly unexpected, as these species are closely related due to a long history of hybridization and introgression in Europe^36,44,76,106,107^. Therefore, discriminating between these two species poses steep analytical challenges. With this panel, the same two *M. galloprovincialis* individuals (B12 and B42) from MG-2 were again classified as *M. chilensis*, likely due to the absence of the PCR-RFLP *Me15-16* marker. On the other hand, the full multi-locus panel including all five markers was fully concordant with PCR-RFLP *Me15-16*, clearly separating the four species, similar to results for a 49-SNP panel analyzed by Larraín *et al*.^38^. These results indicate that a multi-locus approach, as described here, might improve the accuracy of PCR-RFLP marker-based specimen identification. Furthermore, using a single marker may result in poor performance, especially in mixed populations. The capabilities and limitations of each marker in a mono-locus and multi-locus approaches summarized in Table 2, can be useful when analyzing the results of previous studies in which these markers were applied.

**Table 2:** Summary of marker performance in the specimen identification of *Mytilus* mussels (*M. trossulus*, *M. edulis*, *M. chilensis* and *M. galloprovincialis*).

Marker	Diagnostic level by species			Capable to detect	
	Fully diagnostic	Semi diagnostic	Not diagnostic	F1 hybrids	introgressed individuals
*Me 15-16*	*M. trossulus M. edulis M. chilensis M. galloprovincialis*	—	—	Yes	No
*mac-*1	—	*M. trossulus M. edulis M. chilensis M. galloprovincialis*	—	No	No
*ITS*	*M. trossulus*	—	*M. edulis M. chilensis M. galloprovincialis*	Yes	No
*COI Xba*I	*M. chilensis*	—	*M. trossulus M. edulis M. galloprovincialis*	No	No
*16s rRNA*	*M. trossulus M. chilensis*	*M. edulis M. galloprovincialis*	—	Yes*	No
***4 markers:***					
*mac-1, ITS, COI XbaI, 16S rRNA*	*M. trossulus M. chilensis*	*M. edulis M. galloprovincialis*	—	Yes	Yes
***All markers:***					
*Me 15-16, mac-1, ITS, COI XbaI, 16s rRNA*	*M. trossulus M. edulis M. chilensis M. galloprovincialis*	—	—	Yes	Yes

Fully diagnostic: Characteristic allele or haplotype fixed in the species and absent in the others.

Semi diagnostic: Characteristic allele or haplotype not fixed in the species and present in the others.

Not diagnostic: No characteristic allele or haplotype allowed to differentiate the species.

*Only in males.

We conclude that the PCR-RFLP markers *Me15-16*, *ITS* and *COI* produce largely equivalent results when applied using a mono-locus approach; however, the latter two are useful only for separating *M. trossulus* and *M. chilensis* from the three other species (Table 2). *Me15-16* distinguished among the four species tested but, as expected form single locus data, was not able to detect introgression. Mono-locus results for the nuclear *mac-1* and mitochondrial *16S rRNA* markers, due to their semi-diagnostic status, were difficult to interpret and showed low concordance with the results derived from the multi-locus approach. All five markers used simultaneously in a multi-locus approach produced more reliable and robust identifications, outperforming each of the markers when used separately, and comparable performance of SNPs panels. These findings support the use of a multi-locus approach when studying this important marine resource, with implications for research on food quality and safety, sustainable production, biodiversity and conservation.

## Supplementary information

Supplementary Information S1-S6
