# Supplementary material for: Comparison between single and multi-locus approaches for specimen identification in Mytilus mussels
Source: Sci Rep. 2019 Dec 23;9:19714. doi: 10.1038/s41598-019-55855-8 (PMC6928075; doi:10.1038/s41598-019-55855-8)
Supplement: Supplementary file 1 — Supplementary Information S1-S6 [file 41598_2019_55855_MOESM1_ESM.pdf]

Comparison between single and multi-locus approaches for specimen identification in *Mytilus* mussels

María Angélica Larraín[1*], Pía González[1+], Claudio Pérez[2+], Cristián Araneda[1,2]

[1] Food Quality Research Center, Departamento de Ciencia de los Alimentos y Tecnología Química, Facultad de Ciencias Químicas y Farmacéuticas, Universidad de Chile, Santiago, Chile

[2] Laboratorio de Genética y Biotecnología en Acuicultura, Departamento de Producción Animal, Facultad de Ciencias Agronómicas, Universidad de Chile, Santiago, Chile

* Corresponding author: mlarrain@uchile.cl
[+] These authors contributed equally to this work.

S1 - Sample information: Species according RFLP-PCR Me15-16 *Aci* I, code, number of individuals, location and collection date.

**Species according RFLP-PCR Me15-16 *Aci* I**	**Code**	**Nº individuals**	**Location**	**Latitude**	**Longitude**	**Date**
M. trossulus	MT-1	50	West Vancouver - Canada	49.3400	-123.2329	05.30.2017
M. edulis	ME-1	50	Prince Edward Island - Canada	46.1002	-63.1447	07.14.2011
	MG-1	49	Galicia - Spain	42.5670	-8.8472	06.20.2008
M. galloprovincialis	MG-2	50	Dichato - Chile	-36.5234	-72.9331	06.13.2008
	MCh-1	50	Putemun - Chile	-42.4316	-73.7499	06.13.2008
M. chilensis	MCh-2	49	Quillaipe - Chile	-41.5487	-72.7707	06.24.2009

S2 - 49 SNPs panel assignment performance

Species according RFLP-PCR *Me15-16 Aci* I	[%]	[Number of individuals]				
		M. trossulus	*M. edulis*	*M. galloprovincialis*	*M. chilensis*	TOTAL
M. trossulus	100.0	17	0	0	0	17
M. edulis	100.0	0	27	0	0	27
M. galloprovincialis	100.0	0	0	105	0	105
M. chilensis	99.5	0	0	1	188	189
GLOBAL	99.7	17	27	106	188	338

Data from

Larraín, M. A., Zbawicka, M., Araneda, C., Gardner, J. P. A. & Wenne, R. Native and invasive taxa on the Pacific coast of South America: Impacts on aquaculture, traceability and biodiversity of blue mussels (Mytilus spp.). Evol. Appl. 11, 298–311 (2018).

S3 - Allele frequencies on locus *mac*-1

Species according RFLP-PCR *Me15-16 Aci* I		*M. trossulus*	*M. edulis*	*M. galloprovincialis*		*M. chilensis*	
	**Code**	**MT-1**	**ME-1**	**MG-1**	**MG-2**	**MCh-1**	**MCh-2**
**Allele size [bp]**	**Location**	**Vancouver**	**P. Edward**	**Galicia**	**Dichato**	**Putemun**	**Quillaipe**
164		0.011	-	-	-	-	-
221		0.011	-	-	-	-	-
251		0.011	-	-	-	-	-
255		0.152	0.260	0.042	0.020	0.330	0.408
266		0.065	0.730	0.521	0.050	0.670	0.582
277		0.054	-	-	-	-	-
287		0.043	-	-	-	-	-
298		-	0.010	0.260	0.640	-	0.010
301		0.011	-	-	-	-	-
303		-	-	0.156	0.070	-	-
328		-	-	0.021	0.220	-	-
370		0.011	-	-	-	-	-
376		0.011	-	-	-	-	-
384		0.011	-	-	-	-	-
409		0.033	-	-	-	-	-
415		0.022	-	-	-	-	-
418		0.011	-	-	-	-	-
420		0.011	-	-	-	-	-
428		0.065	-	-	-	-	-
435		0.011	-	-	-	-	-
466		0.022	-	-	-	-	-
469		0.022	-	-	-	-	-
475		0.054	-	-	-	-	-
478		0.011	-	-	-	-	-
483		0.022	-	-	-	-	-
487		0.141	-	-	-	-	-
494		0.185	-	-	-	-	-

S4 - Assignment performance of markers in a mono and multi-locus approach assigning individuals to species determined by RFLP-PCR *Me15-16 Aci* I

Mono-locus approach

a) *mac*-1

Species according RFLP-PCR *Me15-16 Aci* I	[%]	[Number of individuals] *M. trossulus*	*M. edulis*	*M. galloprovincialis*	*M. chilensis*	TOTAL
M. trossulus	76.1	35	3	0	8	46
M. edulis	62.0	0	31	1	18	50
M. galloprovincialis	67.7	0	19	44	2	65
M. chilensis	37.0	0	57	1	34	92
GLOBAL	56.9	35	110	46	62	253

b) *ITS*

Species according RFLP-PCR *Me15-16 Aci* I	[%]	[Number of individuals] *M. trossulus*	*M. edulis* / *M. galloprovincialis* / *M. chilensis*	TOTAL
M. trossulus	100.0	50	0	50
M. edulis<br>*M. galloprovincialis*<br>*M. chilensis*	100.0	0	208	208
GLOBAL	100.0	50	208	258

c) *CO* I

Species according RFLP-PCR *Me15-16 Aci* I	[%]	[Number of individuals] *M. trossulus* / *M. edulis* / *M. galloprovincialis*	*M. chilensis*	TOTAL
M. trossulus<br>*M. edulis*<br>*M. galloprovincialis*	98.8	164	2	166
M. chilensis	100.0	0	92	92
GLOBAL		164	94	258

d) *16S*

Species according RFLP-PCR *Me15-16 Aci* I	[%]	[Number of individuals] *M. trossulus*	*M. edulis*	*M. galloprovincialis*	*M. chilensis*	TOTAL
M. trossulus	100.0	45	0	0	0	45
M. edulis	98.0	0	49	0	1	50
M. galloprovincialis	7.6	0	59	5	2	66
M. chilensis	100.0	0	0	0	92	92
GLOBAL		45	108	5	95	253

Multi-locus approach

e) *mac-1, ITS, CO* I, *16S*

Species according RFLP-PCR *Me15-16 Aci* I	[%]	[Number of individuals] *M. trossulus*	*M. edulis*	*M. galloprovincialis*	*M. chilensis*	TOTAL
M. trossulus	100.0	50	0	0	0	50
M. edulis	96.0	0	48	1	1	50
M. galloprovincialis	65.2	0	21	43	2	66
M. chilensis	100.0	0	0	0	92	92
GLOBAL		50	69	44	95	258

f) *Me15-16, mac-1, ITS, CO* I, *16S*

Species according RFLP-PCR *Me15-16 Aci* I	[%]	[Number of individuals] *M. trossulus*	*M. edulis*	*M. galloprovincialis*	*M. chilensis*	TOTAL
M. trossulus	100.0	50	0	0	0	50
M. edulis	100.0	0	50	0	0	50
M. galloprovincialis	100.0	0	0	66	0	66
M. chilensis	100.0	0	0	0	92	92
GLOBAL		50	50	66	92	258

S5 - Concordance between RFLP-PCR *Me15-16 Aci* I and each marker measured through Sensitivity, Specificity and Positive likelihood ratio (95% Confidence interval)

Locus		Species			
		M. trossulus	*M. edulis*	*M. galloprovincialis*	*M. chilensis*
mac- 1	S	0.76 (0.61 - 0.87)	0.62 (0.47 - 0.75)	0.68 (0.55 - 0.79)	0.37 (0.27 - 0.48)
	E	1.00 (0.98 - 1.00)	0.61 (0.54 - 0.68)	0.99 (0.96 - 1.00)	0.83 (0.76 - 0.88)
	LR+	314.21 (19.63 - 5030.74)	1.59 (1.21 - 2.10)	63.63 (15.87 - 255.14)	2.12 (1.38 - 3.27)
ITS Hha I	S	1.00 (0.93 - 1.00)	1.00 (0.98 - 1.00)		
	E	1.00 (0.98 - 1.00)	1.00 (0.93 - 1.00)		
	LR+	413.90 (25.97 - 6596.23)	102.00 (6.47 - 1608.66)		
COI Xba I	S	0.99 (0.96 - 1.00)			1.00 (0.96 - 1.00)
	E	1.00 (0.96 - 1.00)			0.99 (0.96 - 1.00)
	LR+	183.22 (11.55 - 2907.57)			83.00 (20.93 - 329.10)
16s rRNA	S	1.00 (0.92 - 1.00)	0.98 (0.89 - 1.00)	0.08 (0.03 - 0.17)	1.00 (0.96 - 1.00)
	E	1.00 (0.98 - 1.00)	0.71 (0.64 - 0.77)	1.00 (0.98 - 1.00)	0.98 (0.95 - 1.00)
	LR+	413.46 (25.94 - 6589.34)	3.37 (2.71 - 4.20)	30.87 (1.73 - 550.73)	53.67 (17.49 - 164.64)
4 markers: *mac-1, ITS* *CO* I *XbaI, 16s rRNA*	S	1.00 (0.93 - 1.00)	0.96 (0.86 - 1.00)	0.65 (0.52 - 0.76)	1.00 (0.96 - 1.00)
	E	1.00 (0.98 - 1.00)	0.90 (0.85 - 0.94)	0.99 (0.97 - 1.00)	0.98 (0.95 - 1.00)
	LR+	413.90 (25.97 - 6596.23)	9.51 (6.31 - 14.32)	125.09 (17.57 - 890.55)	55.33 (18.03 - 169.81)
All markers: Me 15-16, mac-1, ITS *CO* I *XbaI, 16s rRNA*	S	1.00 (0.93 - 1.00)	1.00 (0.93 - 1.00)	1.00 (0.95 - 1.00)	1.00 (0.96 - 1.00)
	E	1.00 (0.98 - 1.00)	1.00 (0.98 - 1.00)	1.00 (0.98 - 1.00)	1.00 (0.98 - 1.00)
	LR+	413.86 (25.97 - 6595.60)	413.90 (25.97 - 6596.23)	383.12 (24.04 - 6103.63)	326.24 (20.49 - 5193.91)

S: Sensitivity E: Specificity LR+: Positive likelihood ratio

S6 - Haplotype frequencies on locus *16S*

**Species according RFLP-PCR *Me15-16 Aci* I**	***M. trossulus***	***M. edulis***	***M. galloprovincialis***		***M. chilensis***	
**Code:**	**MT-1**	**ME-1**	**MG-1**	**MG-2**	**MCh-1**	**MCh-2**
**Haplotype** / **Location:**	**Vancouver**	**P. Edward**	**Galicia**	**Dichato**	**Putemun**	**Quillaipe**
M. trossulus	0.978	-	-	-	-	-
M. trossulus *	0.022	-	-	-	-	-
M. edulis	-	0.980	0.957	0.750	-	-
M. galloprovincialis (NHG)	-	-	0.043	-	-	-
M. galloprovincialis *	-	-	-	0.150	-	-
M. chilensis (SHG)	-	0.020	-	0.100	1.000	1.000

* New haplotype
